# Features Extraction of Flotation Froth Images and BP Neural Network Soft-Sensor Model of Concentrate Grade Optimized by Shuffled Cuckoo Searching Algorithm

**DOI:** 10.1155/2014/208094

**Published:** 2014-07-16

**Authors:** Jie-sheng Wang, Shuang Han, Na-na Shen, Shu-xia Li

**Affiliations:** ^1^School of Electronic and Information Engineering, University of Science & Technology Liaoning, Anshan 114044, China; ^2^National Financial Security and System Equipment Engineering Research Center, University of Science & Technology Liaoning, Anshan 114044, China

## Abstract

For meeting the forecasting target of key technology indicators in the flotation process, a BP neural network soft-sensor model based on features extraction of flotation froth images and optimized by shuffled cuckoo search algorithm is proposed. Based on the digital image processing technique, the color features in HSI color space, the visual features based on the gray level cooccurrence matrix, and the shape characteristics based on the geometric theory of flotation froth images are extracted, respectively, as the input variables of the proposed soft-sensor model. Then the isometric mapping method is used to reduce the input dimension, the network size, and learning time of BP neural network. Finally, a shuffled cuckoo search algorithm is adopted to optimize the BP neural network soft-sensor model. Simulation results show that the model has better generalization results and prediction accuracy.

## 1. Introduction

Flotation is known as froth flotation, and it is a physicochemical reaction process. Flotation is the process which is based on the differences of the surface property of solid materials to separate useful minerals and gangue by means of the buoyancy of air bubbles from ore pulp by this method to improve the concentrate grade [1]. In the production process of flotation, concentrate grade and other economic and technical indicators are key control indicators of the production process. Process control indicators of domestic flotation process are mainly based on an experienced operator to observe the information (such as foam color, size, flow rate, and texture features) which is provided by the bubble state formed on the surface of the flotation tank and to adjust the flotation level and change agents system. Froth flotation method of manual observation has limitations of its space, time, and subjectivity; meanwhile this method cannot be organically combined with computer control systems to implement advanced control. Inference estimate (soft sensor) technology can effectively solve the online estimation problems that the online measurement of the economic and technical indicators in the flotation process is difficult.

Digital image processing techniques are applied to froth feature extraction and key technical indicators of soft-sensor modeling in the flotation process by scholars at home and abroad and many achievements have been made [2–6]. Woodbum and many other scholars created a bubble dynamic model based on image processing through researching flotation foam structure and calculated the content of useful minerals in foam through this model [7]. Zhou et al. extracted color and size characteristics of the foam by using digital image processing method and established a recovery prediction model, which was proved to have good predictive results by analyzing foam and process image data [8]. Bartolacci et al. extracted visual characteristic parameter from the flotation RGB image by the use of MIA and GLCM methods, and using partial least squares method established predictive model of flotation foam concentrate grade. The model is used in the case of an industrial mineral process and demonstrated the effectiveness of this method [9]. Zhou et al. extracted image feature such as bubbles' color, speed, and size as the model parameters and built predictive model using least squares support vector machine. The experimental results show that this method can effectively predict recovery [10]. Chun-hua et al. established a soft-sensor model of flotation pulp pH value based on sparse multicore least squares support vector machines through taking foam video image characteristics as auxiliary variables and using Schmidt orthogonalization theory reducing multicore matrix [11]. After that, they extracted image features by using color cooccurrence matrix (CCM), and this method was compared with the traditional GLCM extracting the image features method. Li et al. took the feature information of the image as the model input by extracting feature information of foam image, and using the sliding window updating and deviation compensation strategy, respectively, updated model parameters in real time and compensated output. This method achieved soft-sensor modeling of concentrate grade of flotation process [12]. Wang and Zhang proposed a kind of soft-sensor model of economic and technical index based on PCA and ANFIS, and combining PSO algorithm with LSM put forward a new learning process to optimize parameters of ANFIS [13]. Geng and Chai utilized least squares support vector machine to establish soft-sensor model of concentrate grade and tailing grade in the flotation process based on analyzing related influencing factors of concentrate grade and tailing grade of the flotation process technology indicators [14].

The established flotation process soft-sensor model above just used part feature information of the froth image in the flotation process, but much feature information of flotation froth image was not integrated, coordinated, and optimized. In this paper, a BP neural network soft-sensor model based on color feature parameters, visual feature parameters, and shape feature parameters of flotation froth images is proposed, and then a shuffled cuckoo search algorithm based on adaptive step is put forward to optimize BP neural network soft-sensor model parameters. Simulation results demonstrate the effectiveness of the proposed method. The paper is organized as follows. In Section 2, the technique flowchart of the flotation process is introduced. The feature extraction methods of flotation froth images are presented in Section 3. In Section 4, the soft-sensor modeling of flotation process is introduced. The simulation experiments and results analysis are introduced in details in Section 5. Finally, the conclusion illustrates the last part.

## 2. Technique Flowchart of Flotation Process

**Figure 1:**
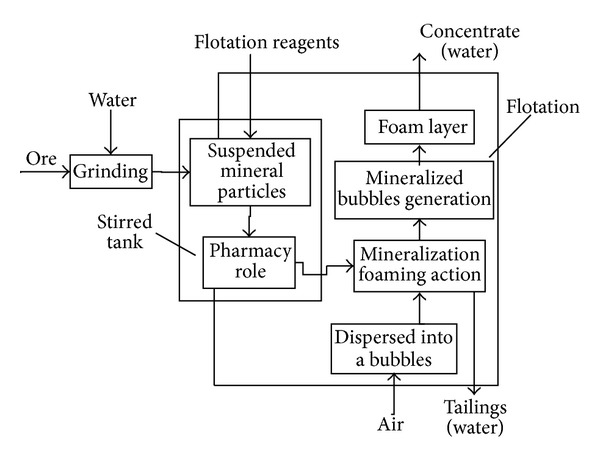
Basic technique diagram of flotation process.

**Figure 2:**
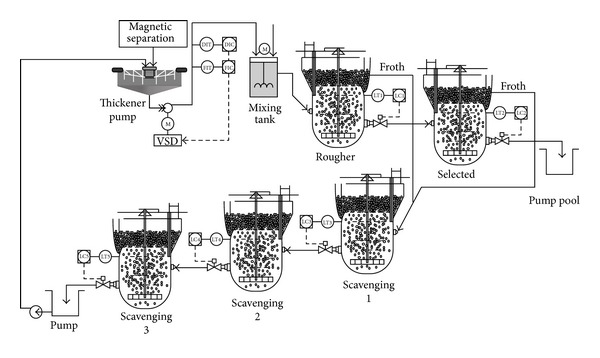
Technique flowchart of flotation process.

Flotation is a sorting mineral technology in the vapor-liquid-solid interface, which consists of roughing, selection, and scavenging process. Flotation is divided into positive flotation and reverse flotation. The so-called positive flotation is that the flotation froth above the flotation tank is concentrate, and the underflow slurry is tailing. Instead of reverse flotation process, the basic principle of positive flotation shows in Figure 1.  Figure 2 shows a typical iron ore flotation process consisting of roughing, selection, and scavenging processes [15].

The input of the system is concentrate pulp under a fine sieve, which is the output of the early front beneficiation process, and the pulp density is about 38%, and concentrate grade is about 64%. The entrance pulp is pumped into the efficient stirred tank through a slurry pipeline. Meanwhile, flotation reagent which is according to a certain concentration ratio is pumped into efficient stirred tank by dosing pump, and pulp temperature reaches suitable temperature through heat treatment. Then the entrance pulp flows into the flotation tank, and if the dose is proper the flotation tank can output concentrate that its grade is 68.5%–69.5%. The output tailings of the flotation tank are fed into scavenger tank for further processing. A part of output of scavenger tank is pumped into an efficient stirred tank for reprocessing by the central mine return pump, and another part of output goes into the postflotation process, magnetic separator, dewatering tank, and other equipment for postprocessing. A part of results is discharged as the tailings, and another part is pumped into the efficient stirred tank for recycling.

Control objective of flotation process is to ensure that the concentrate grade and tailings recovery are controlled within a certain target range. So using the way of offline artificial test obtains grade value, and then, according to the grade value, operators adjust the flotation cell liquid level and the amount of flotation reagents. Because artificial test is 2 hours once; thus, when the process variables and boundary conditions of flotation process change, operators cannot adjust flotation cell liquid level and pharmaceutical dosage based on test values timely, which often results in that flotation concentrate grade and tailings recovery are too high or too low. So it has important industrial application values adopting soft-sensor modeling method to achieve real-time measurement of concentrate grade for optimal control of flotation process.

## 3. Feature Extraction of Flotation Froth Images

Bubble image contains important information which is related to the flotation process, and traditionally the flotation froth state is observed by experienced workers. Generally, flotation froth state can be described by bubble size, foam color (gray value), texture, and stability. However, due to the subjectivity of the operators, understanding of flotation froth state is different and bubble state cannot be observed carefully, which result in that flotation process control cannot reach the optimal state. Therefore, the use of digital image processing technology analyses and processes bubble image, which has important implications for predicting flotation performance indicators.

### 3.1. Digital Image Processing Technology

Digital image processing, also known as the computer image processing, refers to convert image signal into digital signal and use the computer to deal with the process; that is to say, it makes traditional analog image store as digital image by image sampling and quantification [16]. The digital image acquisition system comprises three basic units: the imaging system, the sampling system, and the quantizer. Sampling is a quantifying process of spatial coordinates actually, and quantization is a discrete process of the function value of the image. Sampling and quantizing system are collectively referred to as the digitization. Pixel is the basic element of a digital image, and each pixel has position coordinates with the integer row (high) and column (wide). In the sampling, if the number of horizontal pixels is *m*, the number of vertical pixels is *n*, and the total number of pixel is *m* × *n* pixels in the image. The number of different gray values is referred as gray level in the image. Generally an image has 256 gray levels. If the storage size of an image is *m* × *n* × 8, then the size of the image data is got. A *m* × *n* digital image can be expressed as a matrix:

(1) $$\begin{array}{c} F=\left[\begin{array}{cccc} f\left(0,0\right) & f\left(0,1\right) & \cdots & f\left(0,n-1\right)\\ f\left(1,0\right) & f\left(1,1\right) & \cdots & f\left(1,n-1\right)\\ \vdots & \vdots & \vdots & \vdots \\ f\left(m-1,0\right) & f\left(m-1,1\right) & \cdots & f\left(m-1,n-1\right) \end{array}\right]. \end{array}$$

**Figure 3:**
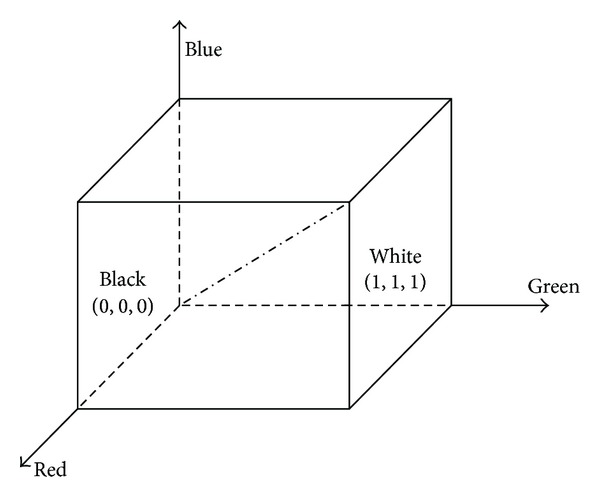
RGB color space model.

Each value of matrix corresponds to a pixel of a digital image; digital image represented as matrix form has the advantage of analysis of bubble images, and thus we can obtain more information about the flotation process. In the computer, according to the number of colors and gray scale, images can be divided into four basic types: binary image, gray image, indexed image, and true color RGB image. True color RGB image refers to that each pixel value is divided into the R, G, and B, three primary color components, and each primary color component will directly determine the intensity of its color. RGB color model produces a variety of different colors by different degrees superposition of the three primary colors. This standard can cover all the colors the human eyes can perceive, and it is one of the widely used color system currently. RGB color space can be represented by a three-dimensional Cartesian coordinate system as shown in Figure 3.

Generally, flotation froth image is a true color RGB image; each pixel is specified by the three values in the image: red (R), green (G), and blue (B) of the three color components. This is represented by a three-dimensional array, namely, *m* × *n* × 3 array. Because the red, green, and blue components are, respectively, represented by 8-bit gray levels; that is to say, (R, G, B)∈[0,…, 255], so the image can contain 2^24^ kinds of color theoretically. Since any color can be obtained by mixing red (R), green (G), and blue (B), the three primary colors, therefore the image can be represented by three-dimensional function of position coordinates:

(2) $$\begin{array}{c} f\left(x,y,z\right)=\left\{f_{\text{red}}\left(x,y,z\right),f_{\text{green}}\left(x,y,z\right),f_{\text{blue}}\left(x,y,z\right)\right\}, \end{array}$$

where *f* represents the position point color of space coordinate (*x*, *y*, *z*) and *f*_red_, *f*_green_, and *f*_blue_, respectively, represent component values of red, green, and blue of this position point. But a planar image is usually considered in study; since each point only includes two coordinate values on the plane, a planar image can be expressed by the two-dimensional function of the position coordinates:

(3) $$\begin{array}{c} f\left(x,y\right)=\left\{f_{\text{red}}\left(x,y\right),f_{\text{green}}\left(x,y\right),f_{\text{blue}}\left(x,y\right)\right\}, \end{array}$$

where two-dimensional function *f*(*x*, *y*) represents a pixel of an image, namely, each value in (1).

RGB color model is very simple, but since this model is uneven from the point of view of human perception and its three components have a larger correlation, we rarely apply RGB space model of images directly. HSI color space is based on the human visual system, and using hue, saturation (chroma), and brightness (intensity) describes color. RGB color space can be converted to the HSI space by transforming.

### 3.2. Image Feature Selection of Flotation Froth

**Figure 4:**
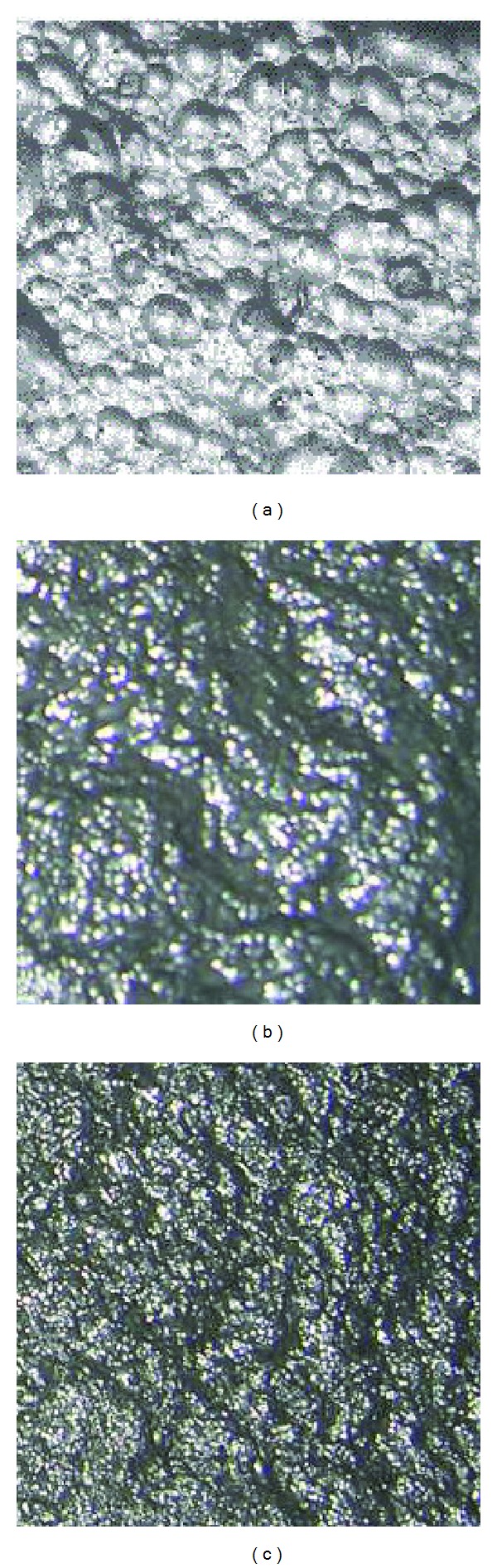
Typical iron ore flotation froth images.

Flotation froth image is got from a CCD camera mounted on a flotation cell. Then the computer image acquisition card converts the continuous analog signals into discrete digital signals, and discrete digital signals are sent into computer for flotation froth visual feature extraction. Typical flotation froth images are shown in Figure 4.

According to flotation technology and expertise, froth image will be divided into three categories as follows. (1) The size of bubbles is larger, and there are parts of large bubbles in the foam. The texture is lighter, but rough. The image complexity is small, and the color is hoary. The foam contains less SiO_2_, and the grade of ore concentrate is low. (2) The bubble size is appropriate, uniform, and stable, the color is partial gray, the texture is finer, and image is more complex. At this time, flotation process is good, and iron concentrate grade meets the requirements. (3) The color of foam is darker, even partial black. The foam is finer, some foam is even difficult to distinguish, and the texture is very complex. At this time, the foam has a high SiO_2_ content. Although iron concentrate grade of output is higher, the drug dosage is too big; therefore, this does not meet the economic requirements of companies.

In the flotation process, the foam layer and flotation performance indexes have important links [17, 18], such as color and froth size. It is important for predicting the performance indexes of the flotation process to extract feature information of flotation froth image. Feature extraction is a concept of computer vision and image processing. It refers to that using a computer extracts image information and determines whether each point of the image belongs to an image feature. The results of the feature extraction are to divide points of image into different subsets. These subsets are often isolated points, a continuous curve, or a continuous area. In the froth flotation process, flotation foam is discharged from flotation tank by the movement of a mechanical scraper. The moving speed of the scraper has a great influence on the mobility of the foam. So the measuring difficulty of the foam mobility is larger. Therefore, we can first consider the image color features and texture features. The shape is the outline of the object, and it does not change with the target color of images. Hence, the shape feature of images also has the advantages which the color and texture features do not have, and it is necessary to consider the shape feature of the image. Therefore, color feature, texture feature, and shape feature of froth flotation images are extracted in this paper.

### 3.3. Color Feature Extraction of Flotation Froth Images

**Figure 5:**
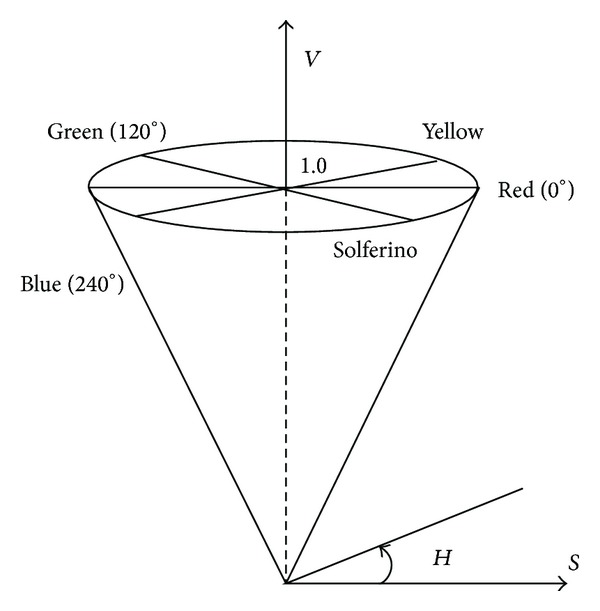
HSI color space model.

Color is a very important visual feature for images, and it has certain stability. It is not sensitive for the changes of size, orientation, translation, and rotation of the image. The operator of flotation production process is also based on the closeness between flotation froth color and gray to determine the merits of the flotation process. System collected images are RGB true color images, which are indicated by red, green, and blue, but the three components often have close correlation. Because the sensitivity to brightness of the human visual is far stronger than the sensitivity to the color shades, the color information of Hue, Saturation and Intensity (HSI) model is similar to the human color visual perception. In order to dispose and identify color conveniently, the human visual system has often used HSI color space, which is more in accord with the human visual characteristic than RGB color space. In the image processing and computer vision, a lot of algorithms can be easily used in the HSI color space; meanwhile they can be treated separately and are independent of each other. Therefore, image analysis and processing workload can be greatly simplified in the HSI color space. HSI color space can be described using a cone-space model.  Figure 5 is the HSI color space model.

In HSI model, hue (*H*) can be measured from 0 to 360 degrees, which represents feels of human senses for different colors, such as red, green, and blue. It may also represent a range of colors, such as warm color and cool color; wherein the angle of pure red is 0, the angle of pure green is 2*π*/3 and the angle of pure blue is 4*π*/3. Saturation (*S*) is the distance between any point in the color space and *I* axis, which represents the depth of color or the degree of shade. Intensity (*I*) is mainly affected by the light source, which represents the light and dark degree of colors. In industrial applications, the range of *S* is [0,1], which changes from unsaturation to a full saturation (nonwhite); the range of *I* is [0,1], which corresponds to color from dark to bright. HSI color space and RGB color space are just different representations of the same physical quantity, so there is a conversion between them:

(4) I=R+G+B3,S=1−3R+G+B[min⁡(R,G,B)],H=cos⁡−1[(R−G)+(R−B)2(R−G)2+(R−B)(G−B)]R≠B or G≠B.

Thus, the three characteristic parameters (hue, saturation, and brightness) extracted from images are as model inputs to predict the content of the concentrate grade.

### 3.4. Texture Feature Extraction of Flotation Froth Image

Image texture reflects the attribute of image itself. In general, texture is a pattern with a small shape and arranged regularly in a certain range of an image. It is an important characteristic for describing images. For the flotation industrial production process, flotation froth images appear to be more obvious texture features, and their texture statistical characteristics can reflect the flotation process conditions. Statistics method, structure method, and spectral method are three texture analysis methods. For different situations, we should adopt different analysis methods. When texture is subtle, for example, wood, meadows, forests, and so forth, statistical analysis method can be adopted. When texture is coarse and has a strong regularity, structural analysis method can be adopted; spectrum method refers to using the frequency characteristics of Fourier transform to describe the periodic texture images.

**Figure 6:**
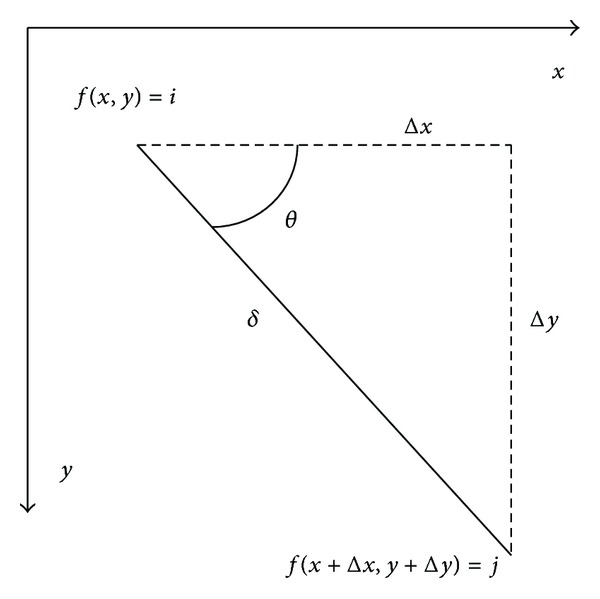
Grey-level cooccurrence matrix.

As can be seen in Figure 4, texture of flotation process froth images is relatively subtle, so we adopt statistical analysis method. In the statistical analysis method, the frequently used methods are histogram analysis method and GLCM method (GLCM). Although the histogram is relatively simple, intuitive, the histogram is a measure of similarity; that is to say, images may have different texture features even though they are similar. The texture is formed by the gray distribution recurring in the spatial position; therefore, there will be a certain gray relationship between two pixels separated by a certain distance in the image space, namely, the gray spatial correlation properties in images. Gray level cooccurrence matrix (GLCM) is a common way describing the texture by studying the spatial correlation properties of gray, which is based on the second-order combination condition probability density function of estimation images. Cooccurrence matrix is defined using the joint probability density of pixels of two positions. It not only reflects the distribution characteristics of intensity but also reflects position distribution characteristics of pixels that are with the same or near intensity. It is the second-order statistical characteristics related image intensity changes, and it is also the basis for the definition of a set of texture features.  Figure 6 is a GLCM schematic view, wherein *i*, *j* indicate the corresponding pixel gray values.

Supposing *f*(*x*, *y*) is a two-dimensional digital image, GLCM means the simultaneous occurrence probability *P*(*i*, *j*, *δ*, *θ*) of two pixels. They are the pixel with gray scale *i* from the image *f*(*x*, *y*)and the pixel (*x* + Δ*x*, *y* + Δ*y*) with gray scale *j*, declination *θ* and distance *δ*. The mathematical formula is expressed as:

(5) P(i,j,δ,θ) ={[(x,y),(x+Δx,y+Δy)]    ∣ f(x,y)=i,  f(x+Δx,y+Δy)=j;    x=0,1,…,Nx−1;  y=0,1,…,Ny−1},

where *i*, *j* = 0,1,…, *L* − 1, *x*, *y* are the coordinates of pixels in the image, *L* is the image gray levels, and *N*_*x*_, *N*_*y*_ are the number of rows and columns of the image, respectively. According to the above definition, the *i*th row and *j*th column elements of the composed gray cooccurrence matrix mean the appearing frequency of all pairs of pixels that with direction *θ*, separation *δ*, gray *i* and *j*, respectively.

In order to describe texture condition using the cooccurrence matrix intuitively, we do not directly use cooccurrence matrix generally but derive some parameters that can reflect the matrix situation from the cooccurrence matrix. These parameters can describe texture features from different angles. GLCM has rich feature parameters, so that it can describe the texture from different angles. Haralick et al. [19] have proposed 14 kinds of texture feature parameters calculated from GLCM. In this paper, four texture characteristic parameters based GLCM such as angular second moment (energy, *f*_1_), contrast (inertia moment, *f*_2_), entropy (*f*_3_), and correlation (*f*_4_) are used to describe the visual characteristic parameters in the flotation froth image:

(6) f1=∑i=1L ∑j=1L{P(i,j)}2,f2=∑n=0L−1n2{∑i=1L ∑j=1L|i−j|=nP(i,j)},f3=−∑i=1L ∑j=1LP(i,j)log⁡{P(i,j)},f4=∑i=1L∑j=1L(ij)P(i,j)−μxμyσxσy.

### 3.5. Shape Feature Extraction of Flotation Froth Image

The shape is the outline of the object; it does not change with the target color in images. It is an important feature of an object. If the image is viewed from different angles, the shape feature of the image may vary. Thus, if shape feature of the image need to be described accurately, it is necessary to make the image have constant characteristics such as scaling, rotation transformation, and translation. There are two methods of the shape feature of the image in general: shape feature representation based profile and feature representation based region. Features representation based profile is suitable for the images that can be obtained easily and their edges are clearness. And shape feature representation based regional is suitable for a single color image. It can be seen from the flotation foam images that feature representation based region can be adopted. Therein, the shape features of the object can be described with rectangularity, circularity, invariant moment, and skeleton [20].

When the geometry of the object is relatively simple, using rectangularity and circularity to describe the shape is more appropriate. However, if boundary feature of the image is more complex, it is more difficult to use these two parameters to describe the shape. So for complex objects, invariant moment can be used to describe shape features of images. Moment characteristics mainly represent the geometric characteristics of the image area, also known as geometric moment. Because it has the constant characteristics such as rotation, translation, scaling and other characteristics, it is also known as the invariant moment. Geometric moment [21] is raised by Hu (visual pattern recognition by moment invariants) in 1962. (*p* + *q*) orders geometric moment of the image defines as follows:

(7) $$\begin{array}{c} m_{p,q}=\overset{+\infty }{\underset{-\infty }{\int }}\overset{+\infty }{\underset{-\infty }{\int }}x^{p}y^{q}f\left(x,y\right)dx\;dy\;\left(p,q=0,1,\ldots \right), \end{array}$$

where *f*(*x*, *y*) is the gray of the image. Since *m*_*p*,*q*_ does not have the translation invariance, define *p* + *q* orders central moment as follows:

(8) vpq=∫−∞+∞∫−∞+∞(x−x¯)p(y−y¯)qf(x,y)dx dy(p,q=0,1),

where $\bar{x},\bar{y}$ is the centroid:

(9) $$\begin{array}{c} \bar{x}=\frac{m_{10}}{m_{00}},\;\;\;\bar{y}=\frac{m_{01}}{m_{00}}. \end{array}$$

*v*_*pq*_ only has translation invariance. In order to get a telescopic invariant moment, define the normalized moment as follows:

(10) $$\begin{array}{c} \mu_{pq}=\frac{v_{pq}}{v_{00}^{1+(p+q)/2}}. \end{array}$$

In this case, the normalized moment has invariant moment when it is translated, stretched or rotated. Hu gives seven invariant moments Φ_1_ − Φ_7_:

(11) Φ1=μ20+μ02,

(12) Φ2=(μ20−μ02)2+4μ112,

(13) Φ3=(μ30−3μ12)2+(3μ21−μ03)2,

(14) Φ4=(μ30+3μ12)2+(μ21+μ03)2,

(15) Φ5=(μ30−3μ12)(μ30+3μ12)×[(μ30+μ12)2−3(μ21+μ03)2]+(3μ21−μ03)(μ21+μ03)×[3(μ30+μ12)2−(μ21+μ03)2],

(16) Φ6=(μ20−μ02)[(μ30+μ12)2−(μ21+μ03)2]+4μ11(μ30+μ12)(μ21+μ03),

(17) Φ7=(3μ21−μ03)(μ30+μ12)×[(μ30+μ12)2−3(μ21+μ03)2]−(μ30−3μ12)[3(μ30+μ12)2−(μ21+μ03)2].

## 4. Soft-Sensor Modeling of Flotation Process

### 4.1. Structure of Soft-Sensor Model

**Figure 7:**
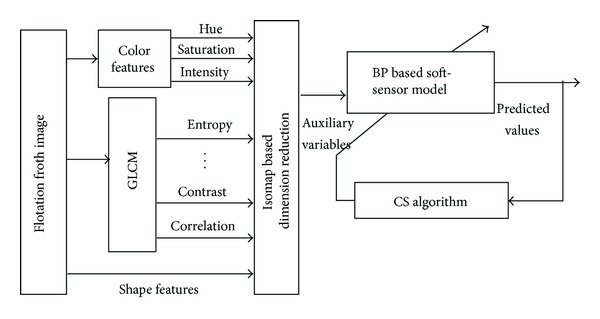
Structure of flotation soft-sensor model.

In this paper, take the texture features, color features, and shape features extracted from the flotation froth images as auxiliary variables of soft-sensor model. Isometric mapping will be carried out to reduce dimensions of auxiliary variables and reduce the target dimensions of BPNN and neural network scale. Finally, using an improved cuckoo search algorithm optimizes BP neural network weights and thresholds to achieve accurate prediction of flotation concentrate grade. The structure of BPNN soft-sensor model optimized by the improved cuckoo search algorithm proposed by this paper is shown in Figure 7.

For a multi-input single-output (MISO) system, the training set can be represented as *D* = {*Y*, *X*_*i*_∣*i* = 1,2,…, *m*}. Where *Y* denotes the output, *X*_*i*_ denotes the *i*th input vector, and it can be represented as *X*_*i*_ = [*x*_1*i*_,*x*_2*i*_,…,*x*_*ni*_]′ (*n* is the number of the training sample sets, *m* is the number of the input variables). Establishment of soft-sensor model needs a data set from the normal condition as modeling data. Assuming the *m* process variables, *n* data vector samples comprise testing data matrix *X* ∈ *R* ^*n*×*m*^. In order to avoid the effects of different dimensions of process variables on predictive results and realize easy mathematical handling, it is necessary to normalize the data. Assuming mean vector of *X* is *μ*, standard deviation vector is *σ*. After the normalization, process variables are

(18) $$\begin{array}{c} \hat{X}=\frac{\left(X-\mu \right)}{\sigma }. \end{array}$$

Then the input vector $\hat{X}$ of the training sample are brought into BPNN to get predictive output $\hat{Y}$, and the root-mean-square error (RMSE) is adopted as the fitness value of soft-sensor model:

(19) $$\begin{array}{c} \text{RMSE}=\sqrt{\overset{n}{\underset{k=1}{\sum }}\frac{{\left({\hat{Y}}_{k}-Y_{k}^{*}\right)}^{2}}{n}}, \end{array}$$

where Y* is the actual output of the training sample.

### 4.2. Data Dimensionality Reduction Based on Isometric Mapping Method

In this paper, 3 colors characteristic parameters, 4 texture characteristic parameters, and 7 shape characteristic parameters extracted from flotation froth images are taken as inputs of BP soft-sensor model, and concentrate grade is as output. Thus, a multi-input single-output soft-sensor model is built. Through scene collecting a batch of flotation foam image features values and concentrate grade measured values of corresponding period from the flotation operations process we can build the soft-sensor model; the input and output data sets are shown in Table 1.

Input and output data sets containing 14 feature variables extracted from the flotation froth images are as auxiliary variables of soft measurement model. It realizes prediction of concentrate grade in flotation process, but there are some problems of jumbled information, repeated expression, and so on. And for BPNN, the input vector dimension is too large, which will make the network topology and training become very complex, so in this paper isometric mapping method is used to reduce the dimensionality of high dimensional input vectors [22]. Isometric mapping is a technique that can solve the problem of saving geodesic distance between data points. It can achieve the dimension reduction process of high-dimensional vectors. In this paper, Isomap algorithm is applied to feature information extracted from foam images to reduce dimension of high-dimensional input data and get a four-dimensional data set, taking the data set as inputs of soft-sensor model, which simplifies the model scale and improves operational efficiency.

Isomap algorithm is based on MDS algorithm. MDS algorithm is a classic method of remaining Euclidean distance unchanged theoretically, which is the earliest visualization operation applied to data. The algorithm is based on the idea that distant points in the high-dimensional space are still far away in a low-dimensional embedding space; while adjacent points in the high-dimensional space remain in adjacent positional relationship in a low-dimensional space. Its data characteristic is not the specific position of each point but is the differences between them. The differences generally use a distance to measure; that is to say, the more similar the two data points, the smaller the distance between them. The commonly used distances are Euclidean distance, Manhattan distance, Minkowski distance, and so no. When we use Euclidean distance, MDS and PCA are equivalent.

Isomap algorithm seeks to maintain inherent geometric properties of data points, namely, maintaining the geodesic distance between two points. The greatest difference between Isomap and MDS is that the distance matrix structured by MDS reflects the Euclidean distance between sample points, while distance matrix structured by Isomap reflects the geodesic distance between sample points. Therefore, the key step of Isomap is how to calculate geodesic distance between sample points. There are many ways of estimating geodesic distance; the classic Isomap method is as follows: for each data point *x*_*i*_, find *k* adjacent points, then the geodesic distance between *x*_*i*_ and *k* adjacent points is approximated by the Euclidean distance between them. The geodesic distance between other nonadjacent points is accumulated by the shortest path between adjacent points. The calculation algorithm is as follows.

**Step 1 (Select neighborhood).**

Structure neighborhood graph *G* of the data set *X*. Calculate Euclidean distance between each sample point *x*_*i*_ and other sample points. When *x*_*j*_ is one of the *k* nearest neighbor points of *x*_*i*_, then setting the weight of the adjacent edge is *d*(*i*, *j*).

**Step 2 (Calculate the shortest path).**

When the neighborhood graph *G* has the side from *x*_*i*_ to *x*_*j*_, note the shortest path *d*(*i*, *j*); otherwise *d*(*i*, *j*) = *∞*. For *l* = 1,2,…, *N*, there is *d*_*G*_(*i*, *j*) = min⁡{*d*_*G*_(*i*, *j*), *d*_*G*_(*i*, *l*) + *d*_*G*_(*l*, *j*)}. Thus, get the shortest path matrix *D*_*G*_ = {*d*_*G*_(*i*, *j*)}.

**Step 3 (Calculate D-dimensional embedding).**

{*λ*_1_, *λ*_2_,…, *λ*_*d*_} and *V* = {*v*_1_, *v*_2_,…, *v*_*d*_} are fore *d* eigenvalues and corresponding eigenvectors of matrix *τ*(*D*_*G*_) = −*HSH*/2, respectively. Where *S* represents the shortest path distance squared matrix; that is, *s*_*ij*_ = *d*_*ij*_^2^; *H* is center matrix; that is, *h*_*ij*_ = *δ*_*ij*_ − 1/*N*. Then *Y* = diag⁡{*λ*_1_, *λ*_2_,…, *λ*_*d*_}*V* ^*T*^ is coordinate values of *d*-dimensional coordinate data sets.

### 4.3. BP Neural Network

#### 4.3.1. Structure and Working Principle of BP Neural Network

**Figure 8:**
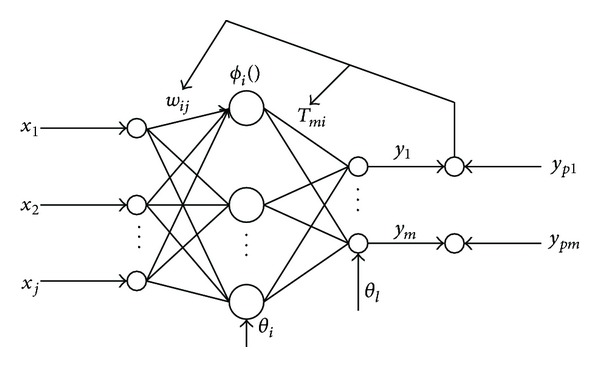
Structure of BP neural network.

Backpropagation neural network is proposed in 1986 by a team of scientists led by Rumelhart and McCelland, which is a multilayer feed-forward network trained by an error back propagation algorithm, and it is one of the most widely used neural network models. Typical BP neural network structure is shown as Figure 8, and the general three-layer structure is *j* − *i* − *m*; that is to say, it has *j* inputs, *i* hidden nodes, and *m* outputs.

In Figure 8, input node is *x* = [*x*_1_,*x*_2_,…,*x*_*j*_]^*T*^, the network weights of input nodes and hidden nodes are *w*_*ij*_, the hidden node is *o* = [*o*_1_,*o*_2_,…,*o*_*i*_]^*T*^, weights of hidden nodes and output nodes are *T*_*mi*_, output node is *y* = [*y*_1_,*y*_2_,…,*y*_*m*_]^*T*^, and the desired output of network is *y*_*p*_. *ϕ*_*i*_(·) is the *i*th hidden node activation function. Usually, *S*-type logarithmic function is served as the activation function of the network, and hyperbolic tangent function can also be used as follows:

(1) *S*−type log function
  (20) $$\begin{array}{c} \phi \left(x\right)=\frac{1}{1+\exp\left(-x\right)}, \end{array}$$
(2) hyperbolic tangent function
  (21) $$\begin{array}{c} \phi \left(x\right)=\frac{2}{1+\exp\left(-2x\right)}-1. \end{array}$$

Working principle of BP neural network consists of two parts: forward propagation and error backpropagation. Each neuron of input layer receives input information from the outside world and transmits to the intermediate layer of each neuron; intermediate layer is an internal information processing layer; it charges information conversion; information is passed from the hidden layer to the output layer of each neuron. After further treatment, a learning forward propagation process is completed. The information processing results are outputted by output layer to the outside world. When the actual output and the expected output do not match, the network enters the errors backpropagation phase. When error through the output layer, weights of each layers are corrected according to gradient descent method and back propagating to the hidden layer and input layer. Cycle of information forward propagation and error back propagation process is a process of constantly adjusting the weights of each layer, and it is also a process of the neural network learning and training. This process continues until the network output error reduces to an acceptable level, or the cycle number reaches the preset given number of learning.

#### 4.3.2. Standard BP Learning Algorithm

BP network learning rules are also known as *δ* learning rules. For a set of given training mode, using training mode network repeats the process of fore-propagation and error backpropagation continuously. When the various training modes meet the requirements, the BP network has learned well. Learning algorithm of BP neural network mainly corrects weights and thresholds of the network.

*(1) Momentum BP Algorithm (MOBP).* Basic BP learning algorithm adjusts the weights only along the direction of error gradient descent at *t*th time and does not consider the direction before *t*th time. So the learning process is usually vibratory and the convergence speed is slow. In order to improve the training speed, a momentum term is added to the weight adjustment formula:

(22) $$\begin{array}{c} \Delta w\left(t\right)=\eta \left[a\Delta w\left(t-1\right)-\left(1-a\right)\delta O\right], \end{array}$$

where *w* is weight matrix, *O* is output vector, *η* indicates learning rate, and *a* ∈ [0,1] represents the momentum factor. Momentum *a*Δ*w*(*t* − 1) defined by formula (16) reflects the previous adjustment process. Wherein *d*_*i*_ = ||*n*_*i*_ − *n*_best_||/*d*_max⁡_ which represents the weight adjustment is only related to the current negative gradient; wherein *n*_*i*_ represents the adjustment of the weight depending on the negative gradient of the last training. This momentum can be seen as a low-pass filter, which can smooth fluctuations in the learning process and can also improve the convergence rate.

In addition, when the error gradient occurs a local minimum, although *n*_best_, but *d*_max⁡_ can make error gradient jump out from a local minimum, and can accelerate the iterative convergence

*(2) Variable-Rate BP Learning Algorithm.* In the gradient descent method, the learning rate has a great impact on the entire training process. If the learning rate is too large, the algorithm may oscillate and make it unstable; if learning rate is too small, convergence is slow and the training time is long. That is to say, the success of training depends on how to choose the learning rate. In the training process, if the rate of learning changes constantly and the algorithm is corrected along error performance surface, the training process will be speeded up. As is shown in the following equation:

(23) $$\begin{array}{c} \text{ste}{\text{p}}_{i}=\text{ste}{\text{p}}_{\min}+\left(\text{ste}{\text{p}}_{\max}-\text{ste}{\text{p}}_{\min}\right)d_{i}, \end{array}$$

where step = *α* is incremental factor, *X*_*i*_^*t*^ is decreased factor, step_max⁡_ is learning rate, and step_min⁡_ is the network output total error performance function of the *d*_*i*_th iteration.

In the training process, trying to make the algorithm stable, meanwhile trying to make learning step big. The learning rate is adjusted accordingly according to the local error surface. When the error tends to the target by the way of reduction, it indicates that correction direction is right, and step can be increased. Therefore, the learning rate is multiplied by the incremental factor *d*_*i*_, so that the learning rate increases; when the error increases beyond the preset value, it indicates that correction is excessive and step should be reduced. Therefore, the learning rate is multiplied by the decreased factor *k*_dec_, so that the learning rate decreases. Round the previous step correction process which makes the error increase.

*(3) LM Learning Algorithm.* LM algorithm is designed to avoid calculating the Hessian matrix when correcting uses approximate second order training rate. When the error performance function has the form of error of sum square, Hessian matrix can be approximated as follows:

(24) $$\begin{array}{c} H=J^{T}J. \end{array}$$

Gradient calculation expression is

(25) $$\begin{array}{c} g=J^{T}e, \end{array}$$

where *J* is Jacobian matrix, *H* is Jacobi matrix which includes network error function for the first derivative of weights and thresholds, and *e* is the network error vector. Jacobian matrix can be calculated by a standard feed-forward network technology, and it is much simpler than the Hessian matrix. LM algorithm uses the above formula approximating Hessian matrix and it is corrected according to the following formula:

(26) $$\begin{array}{c} x\left(k+1\right)=x\left(k\right)-{\left[J^{T}J+\mu J\right]}^{-1}J^{T}e. \end{array}$$

### 4.4. BP Neural Network Optimized by Improved Cuckoo Search Algorithm 

#### 4.4.1. Cuckoo Search Algorithm

Cuckoo search algorithm is a new metaheuristic search algorithm, which is based on the behavior of the cuckoo's nest parasitism and Levy flight behavior of some birds and fruit flies. In 2009, cuckoo search algorithm is proposed by the Yang in Cambridge University and Deb in Raman Engineering University [23–25]. The Cuckoo bird is a kind of very interesting bird; they can not only emit pleasant voice, but also have aggressive reproductive strategy. Most of the cuckoo lay their eggs in other birds' nest, making the host tend to cubs instead of them. If the host found that the eggs are not of their own birth, it will put alien eggs out of the nest or choose to abandon its nest and rebuild a new nest elsewhere. However, some cuckoos choose nests of colors and shapes of their eggs similar with the host's to get the love of the host, which will reduce the possibility of their eggs being abandoned and increase the reproduction rate of cuckoos [22, 26].

Under normal circumstances, each cuckoo only can produce one egg, and the egg in each nest represents a solution; the purpose is to make the potential better solution replace the bad solution in the nest. Obviously, this method can be applied to more complex cases. In these cases, there is not only one egg in each nest. And these eggs represent a set of solutions. In order to better study cuckoo search algorithm, we will use the simplest method, which is only one egg in each nest. In this case, an egg, a nest or a cuckoo is no difference in above. That is to say, each nest corresponds to one egg produced by cuckoo.

For a brief description of the cuckoo search algorithm, the following three ideal rules are adopted to construct the cuckoo search algorithm [27, 28].

1. Each cuckoo produces only one egg each time, and randomly selects the nest to hatch.
2. The best nest will be retained to the next generation.
3. The number of nests is fixed, and the probability of a host finding an alien egg is *Pa* = [0,1]. In this case, the host might push the egg out of the nest or abandon the nest and renest a new location.

Cuckoo algorithm is based on a random walk search method of *Levy* flight. *Levy* flight is a random walk that step size is subject to levy distribution, and the walk direction is subject to a uniform distribution. Based on these rules, the location update formula of cuckoos hunting nests is

(27) $$\begin{array}{c} x_{i}^{\left(t+1\right)}=x_{i}^{t}+\alpha s_{L}, \end{array}$$

where *x*_*i*_^*t*^ represents the position of the *i*th nest at the *t*th generation, *x*_*i*_^*t*+1^ represents the position of the *i*th nest at the (*t* + 1)th generation, *α* is step control volume, and *s*_*L*_ is a vector obeying Levy distribution:

(28) $$\begin{array}{c} L\left(s,\lambda \right)=\frac{\lambda \Gamma \left(\lambda \right)\sin\left(\pi \lambda /2\right)}{\pi }\frac{1}{s^{1+\lambda }}, \end{array}$$

where *s*_0_ > 0 is the minimum step, Γ is a gamma function, and *L*(*λ*) step obeys Levy distribution. However, Levy distribution is generally expressed as follows:

(29) $$\begin{array}{c} L\left(\beta ,\lambda \right)=\frac{1}{\pi }\overset{\infty }{\underset{0}{\int }}\cos\left(ks\right)\exp\left[-\beta {\left|k\right|}^{\lambda }\right]dk. \end{array}$$

There is not any explicit analyzer in the formula (29), and, therefore, it is difficult to obtain a random sample by the formula. The formula (29) can be approximated by the following formula:

(30) $$\begin{array}{c} L\left(\beta ,\lambda \right)=\frac{\beta \lambda \Gamma \left(\lambda \right)\sin\left(\pi \lambda /2\right)}{\pi {\left|s\right|}^{1+\lambda }}. \end{array}$$

When *β* = 1, formula (30) is equivalent to formula (28). Although formula (30) can describe the behavior of a random walk of the cuckoo algorithm, it is not conducive to describing the language of mathematics and also is not conducive to writing programs. Therefore, Yang Xin She and Deb found in the realization of the CS algorithm that using Mantegna algorithm can simulate the random walk behavior of Levy flight. In this CS algorithm, the step size *s* can be expressed as

(31) $$\begin{array}{c} s=\frac{u}{{\left|v\right|}^{1/\lambda }},\;1\leq \lambda \leq 2. \end{array}$$

When *β* = 1, formula (30) is equivalent to formula (28). Although formula (30) can describe the behavior of a random walk of the cuckoo algorithm, it is not conducive to describing the language of mathematics and also is not conducive to writing programs. Therefore, Yang Xin She and Deb found in the realization of the CS algorithm that using Mantegna algorithm can simulate the random walk behavior of Levy flight. In this CS algorithm, the step size *s* can be expressed as

(32) $$\begin{array}{c} s\sim N\left(0,\sigma_{\mu }^{2}\right),\;\;v\sim N\left(0,\sigma_{v}^{2}\right),\\ \sigma_{\mu }={\left\{\frac{\Gamma (1+\beta )\sin(\pi \beta /2)}{\Gamma [(1+\beta )/2]\beta 2^{(\beta -1)/2}}\right\}}^{1/\beta },\;\sigma_{v}=1. \end{array}$$

When *β* = 1, formula (30) is equivalent to formula (28). Although formula (30) can describe the behavior of a random walk of the cuckoo algorithm, it is not conducive to describing the language of mathematics and also is not conducive to writing programs. Therefore, Yang Xin She and Deb found in the realization of the CS algorithm that using Mantegna algorithm can simulate the random walk behavior of Levy flight. In this CS algorithm, the step size *s* can be expressed as follows.

Specific steps of CS algorithm are as follows.

**Step 1 (Initialization).**

Randomly generate *N* nest position *X* ^0^ = (*x*_1_^0^, *x*_2_^0^,…, *x*_*N*_^0^), select the best nest position, and retain it to the next generation.

**Step 2 (Search operation).**

Using location update formula search the next nest position and obtain a set of new position. Test these positions and compare them with the previous generation position to get a better position.

**Step 3 (Select operation).**

Generate random numbers *r* ∈ [0,1] which obey uniform distribution, and compare the random numbers with detection probability *Pa* = 0.25. If *r* > *Pa*, change *x*_*i*_^(*t*+1)^ randomly, otherwise unchanged. Test the changed nest position, and compare it with the nest position obtained in the previous step; select the best nest position *pb*.

**Step 4 (Judgment operation).**

Calculate the fitness and judge whether it can reach the required accuracy or iteration termination condition. If reached, *pb* is the optimal solution; otherwise return to Step 2 to continue the iterative update.

#### 4.4.2. Dynamical Adjusting Step

In the basic CS algorithm, the step generated by Levy flight is various. Formula (31) shows that Levy flight entirely depends on the size of the random numbers *u* and *v*; randomness is strong. In the beginning of the search, the value from the search may be far away from the optimal values, which need a large step to reach the optimum value. The bigger the step size, the wider the scope of the search. Thus, it is easier to search the global optimum, but meanwhile it also reduces the search accuracy; with the increase of the iteration, most nests may be close to the optimal value, which need a smaller step. However, when the step size is too small, the search scope becomes dense. Despite the fact that the accuracy of the solution is increased, the search speed is reduced at the same time. Therefore, the step of the conventional Levy flight has a strong randomness and is lack of adaptability. For this case, if we dynamically adjust the step size according the search results of different stages, we can balance global optimization and optimization speed, so that both of them can achieve better results. First introduced

(33) $$\begin{array}{c} d_{i}=\frac{||n_{i}-n_{\text{best}}||}{d_{\max}}, \end{array}$$

where *n*_*i*_ represents the position of the *i*th nest, *n*_best_ represents the current best nest position, and *d*_max⁡_ represents the maximum distance from the optimal nest position to the other nest positions. According to this formula, we introduce the formula of dynamically adjusting the step:

(34) $$\begin{array}{c} \text{ste}{\text{p}}_{i}=\text{ste}{\text{p}}_{\min}+\left(\text{ste}{\text{p}}_{\max}-\text{ste}{\text{p}}_{\min}\right)d_{i}, \end{array}$$

where step = *α* is the search path that cuckoos randomly search new nest positions from the original nest position *X*_*i*_^*t*^ according to (27) each time; step_max⁡_ and step_min⁡_ represent the minimum step and maximum step, respectively.

According to the above equations (33)-(34), we can dynamically adjust the step size. When the *i*th nest location is close to optimal nest location, *d*_*i*_ is smaller, and step size is also smaller; when the *i*th nest location is far away from optimal nest location, *d*_*i*_ is larger, and step size is also larger. Thus according to the search results of different stages, the step size changes and can be adjusted dynamically, which makes cuckoo algorithm have a better adaptability and improve the global search ability and convergence precision.

#### 4.4.3. Shuffled Cuckoo Search Algorithm (SCSA)

Shuffled cuckoo search algorithm draws on ideas of shuffled frog leaping algorithm [29, 30]. The population is divided into a plurality of memes groups optimized by cuckoo search algorithm, which improves the local search capabilities of the population. The basic idea of the SCSA algorithm is to generate initial population *P* = {*X*_1_, *X*_2_,…, *X*_*N*_} consisting of *N* cuckoos randomly, where *X*_*i*_ = [*x*_*i*1_, *x*_*i*2_,…, *x*_*ik*_] represents the *i*th cuckoo. After generating the initial population, order the individuals according to the fitness, and record the optimal fitness value corresponding cuckoo individual *X*_*g*best_ in population. Then the group is divided into *m* groups, each group contains *n* cuckoos, and satisfy *N* = *m* × *n*. Among them, the first cuckoo is divided into group 1, the second is divided into the group 2, the *m*th cuckoo is divided into group *m*, the (*m* + 1)th cuckoo is divided into group 1 again, and so on. Assume that *M* ^*s*^ is the collection of the *s*th group cuckoos, and the process of allocating cuckoos above can be described by

(35) $$\begin{array}{c} M^{s}=\left\{X_{s+m(l-1)}\in P\;|\;1\leq l\leq n\right\},\;\left(1\leq s\leq m\right). \end{array}$$

Then using cuckoo search algorithm searches each group at the same time, that is, Cuckoos in each group are carried out of the selection, searching, and judgment operation. When each group completes the current search, cuckoos will be remixed and sorted in each group, and cuckoo individual *X*_*g*best_′ corresponding to the best fitness at this time will be rerecorded. Compare with the last best cuckoo individual, record the best cuckoo individual, and divide groups according to the formula (35). Local-search again until blended iterations are reached, and output the best individual, that is, the optimal solution.

#### 4.4.4. Flowchart of Shuffled Cuckoo Search Algorithm

**Figure 9:**
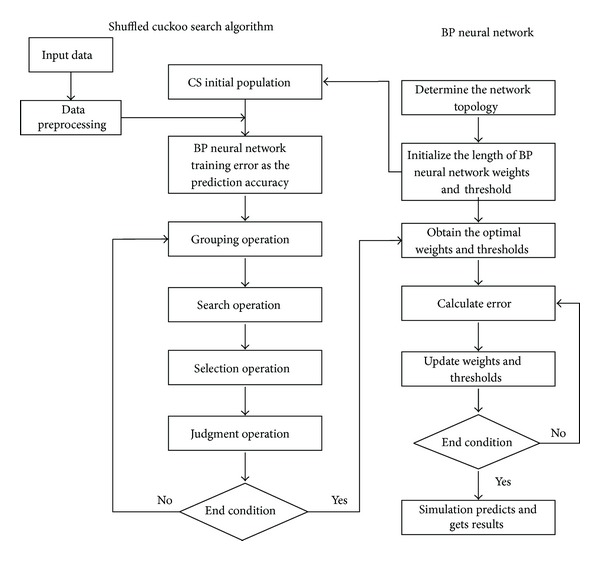
Structure diagram of SCS-BPNN training algorithm.

In this paper, shuffled cuckoo search algorithm is applied to parameters learning of BP neural network soft-sensor model, which aims to improve the local search capabilities to achieve better convergence accuracy. SCSA algorithm is different from the traditional cuckoo algorithm; it divides the initial population into several groups, and then each group is optimized using cuckoo algorithm, respectively. After completing the iteration, the individuals in each group mixed into a population again. Repeat the process above until achieving the number of iterations or the required precision. Because prediction accuracy of BP neural network is related to the initial connections weights and thresholds, if parameters chosen inappropriately can lead to prediction accuracy reduce and fall into local optimum. Therefore, in this paper, adopting this shuffled cuckoo search algorithm to optimize the weights and thresholds of BP neural network establishes BP neural network soft-sensor model optimized by shuffled cuckoo search algorithm. The data of dimensionality reduction acquired from the flotation froth image are taken as inputs of soft measurement model, and concentrate grade is as output of model.  Figure 9 is structure diagram of BP neural network optimized by shuffled cuckoo search algorithm.

The algorithm process is described as follows.

**Step 1 (Parameter initialization).**

Determine the topology of BP neural network, initialize the length of network weight *w* and threshold *b*, and get BP neural network training samples. Set *n* nests, the number of maximum iteration iter_max⁡_.

**Step 2 (Generated population randomly).**

Generate *N* nest locations randomly *X*_*i*_^(0)^ = [*x*_1_^(0)^, *x*_2_^(0)^,…, *x*_*N*_^(0)^]^*T*^, and each nest location corresponds to a set of weights and thresholds of BP neural network. Train BP neural network, calculate the prediction accuracy (the fitness value) corresponding to each group of nests. According to the fitness value, sort the individuals of the population by ascending order and record the current best nest *x*_*b*_^(0)^, *b* ∈ {1,2,…, *N*} and fitness value *fitness*.

**Step 3 (Grouping operation).**

The initial group is divided into *m* groups; each group contains *n* cuckoos and satisfies *N* = *m* × *n*. Among them, the first cuckoo is divided into group 1, the second is divided into the group 2, the *m*th cuckoo is divided into group *m*, the (*m* + 1)th cuckoo divided into group 1, and so on.

**Step 4 (Search operation).**

Retain the previous generation optimal nest location *x*_*b*_^(0)^. Using the position updated formula updates for each of nests, respectively, and a new set of nest position is produced, respectively. Test each of nests and compare them with the previous generation *p* ^(*t*−1)^ = [*x*_1_^(*t*−1)^, *x*_2_^(*t*−1)^,…, *x*_*n*_^(*t*−1)^]^*T*^. Make sure that the better nest location replaces poor nest location, and then each group gets the current best nest position *K*_*i*_^(*t*)^ = [*x*_1_^(*t*)^, *x*_2_^(*t*)^,…, *x*_*n*_^(*t*)^].

**Step 5 (Selection operation).**

For each group of nest locations, the random number obeying uniform distribution *r* ∈ [0,1] serves as the probability of a host finding alien eggs. If *r* > *p*_*a*_, change the location of the nest randomly and get a group of new nest locations. If *r* < *p*_*a*_ does not change the location of the nest, test this group nest location and compare it with the fitness value of each nest location in *k* ^(*t*)^. The nest location which has better fitness value replaces poor fitness value location and obtains a set of new optimum nest locations *p* ^(*t*)^ = [*x*_1_^(*t*)^, *x*_2_^(*t*)^,…, *x*_*n*_^(*t*)^]^*T*^.

**Step 6 (Judgment operation).**

After nest locations of each group experience* i*th iterations, the individuals of all groups are remixed together and sorted according to the fitness value. Record the individual *X*_*g*best_′ corresponding to the best fitness value. Compare it with the optimal nest location *x*_*b*_^(0)^ in Step 4, and the better one serves as the current optimal nest location until reaching maximum iterations iter_max⁡_.

**Step 7 (Model validation).**

The parameters corresponding to the nest optimum position *x*_*b*_^(*t*)^ serve as BP neural network weights and thresholds, retrain the training data, establish BP neural network model, and verify the model using test data.

## 5. Simulation Results

In this paper, the flotation process is as the research object to establish soft-sensor model for the concentrate grade. Firstly, determine 600 groups of input and output data sets of BP neural network soft-sensor model based on SCSA, which is shown in Table 1. Then using isometric mapping method reduces dimension of 600 groups of input and output data sets in Table 1. The processing data serve as inputs of soft-sensor model and the concentrate grade serves as the output of it to establish BP neural network soft-sensor model. The former 550 groups of data are as training data of the model, and the rest of 50 groups of data are as the prediction data of the model. Using improved cuckoo search algorithm proposed by this paper optimizes weights and thresholds of BP neural network. In this paper, select the normalized root mean square error (NRMSE), mean square error (MSE), and mean absolute percentage error (MAPE) and other performance indicators as the basis of judging predictive effects, which is calculated as follows:

(36) $$\begin{array}{c} \text{MSE}=\frac{1}{T}\overset{T}{\underset{t=1}{\sum }}{\left(y\left(t\right)-y_{d}(t)\right)}^{2},\\ \text{RMSE}={\left[\frac{1}{T}\overset{n}{\underset{t=1}{\sum }}{\left(y_{d}\left(t\right)-y\left(t\right)\right)}^{2}\right]}^{1/2},\\ \text{NRMSE}=\sqrt{\frac{1}{T{||y_{d}||}^{2}}\overset{T}{\underset{t=1}{\sum }}{\left(y\left(t\right)-y_{d}\left(t\right)\right)}^{2}},\\ \text{SSE}=\overset{n}{\underset{t=1}{\sum }}{\left(y_{d}\left(t\right)-y\left(t\right)\right)}^{2},\\ \text{MAPE}=\frac{100}{T}\overset{T}{\underset{t=1}{\sum }}\frac{\left|y\left(t\right)-y_{d}\left(t\right)\right|}{y_{d}\left(t\right)}, \end{array}$$

where *T* is the number of predictive sample points, *y*(*t*) is predictive value, and *y*_*d*_(*t*) is actual value of the sample.

**Figure 10:**
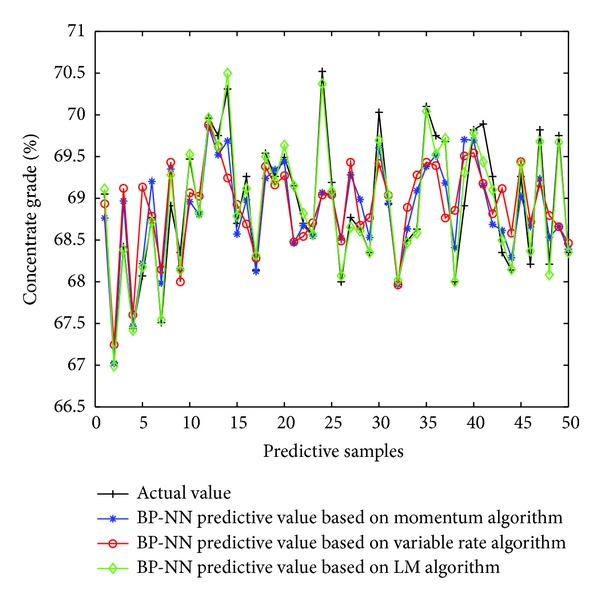
Predictive results of soft-sensor model.

**Figure 11:**
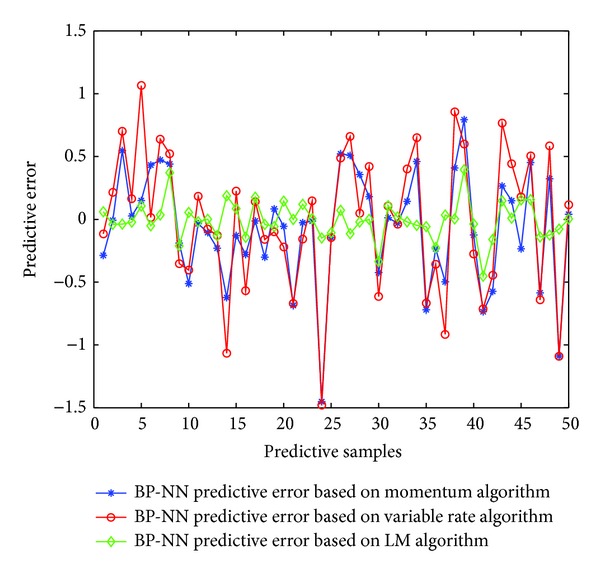
Predictive errors of soft-sensor model.

Firstly, using three learning algorithms of BP neural network described earlier, namely, momentum BP algorithm, variable-rate learning algorithms, and LM learning algorithm, establishes corresponding soft-sensor model, respectively. Then using the three soft-sensor models predicts concentrates grade of the flotation process.  Figure 10 is a comparison of the predictive output and the actual output of the three models, and Figure 11 is the error curve of predictive output of the three models.

**Figure 12:**
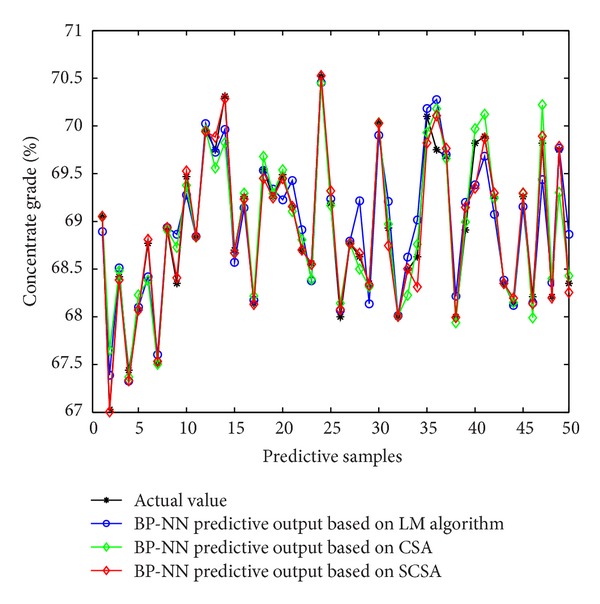
Predictive results of soft-sensor model.

**Figure 13:**
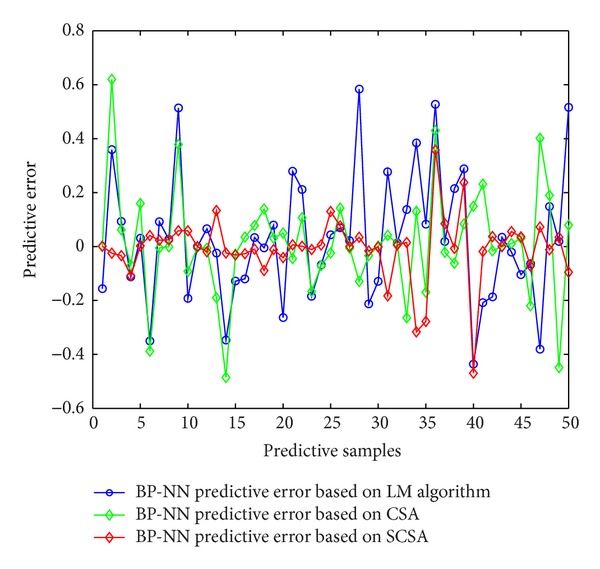
Predictive errors of soft-sensor model.

As can be seen from the predictive output curve and predictive error curve, in the three models, the predictive effects of BP neural network soft-sensor model based on LM algorithm are better. Therefore, in order to prove the effectiveness of shuffled cuckoo algorithm, this paper will compare BP neural network soft-sensor model based on LM with BP neural network soft-sensor model based on SCSA-BP proposed in this chapter. Selecting the latter 50 sets of data from the input and output samples serves as the forecast data of the two models.  Figure 12 is a comparison of the predictive output and the actual output of the two models, and Figure 13 is the error curve of predictive output of the two models. In order to illustrate the advantages of the proposed method better, this paper calculates mean square error, root mean square error, mean absolute percentage error, and other performance indicators of each method. Their prediction accuracy is shown in Table 2. As can be seen from the simulation results, the prediction accuracy of BP neural network soft-sensor model based on the proposed shuffled cuckoo search algorithm is higher than the prediction accuracy of BP neural network soft-sensor model based LM algorithm.

## 6. Conclusions

Through analyzing the froth flotation process image, this paper extracted 14 image feature parameters consisting of texture feature, color feature, and shape feature. And adopting Isomap algorithm reduces dimensionality of high dimensional input vectors, which avoid the disaster of data dimensionality and reduce the complexity of the neural network; the preprocessing data serve as inputs of BP neural network soft-sensor model, and the predictive variable concentrate grade serves as the output of the model. Therefore, the parameters of BP neural network soft-sensor model optimized by an adaptive step shuffled cuckoo search algorithm are proposed. Simulation comparing experimental results verifies the effectiveness of the proposed method.

## Figures and Tables

**Table 1:** Prediction data of soft-sensor model.

Number	Color features			Texture features				Invariant moment (shape features)							Concentrate grade
	Hue	Saturation	Intensity	Energy	Inertia moment	Entropy	Correlation	*n*1	*n*2	*n*3	*n*4	*n*5	*n*6	*n*7	
1	0.234	0.0235	0.446	0.197	0.114	7.117	0.863	0.047	0.183	0.003	0.605	0.312	0.198	0.359	68.28
2	0.286	0.016	0.514	0.203	0.110	13.633	0.834	0.088	0.162	0.012	0.585	0.384	0.189	0.329	67.3
3	0.310	0.019	0.522	0.195	0.110	11.094	0.853	0.085	0.203	0.038	0.618	0.235	0.164	0.391	68.56
4	0.358	0.023	0.502	0.193	0.107	9.850	0.855	0.047	0.197	0.022	0.613	0.236	0.191	0.397	69.26
⋮	⋮	⋮	⋮	⋮	⋮	⋮	⋮	⋮	⋮	⋮	⋮	⋮	⋮	⋮	⋮
600	0.229	0.028	0.461	0.192	0.266	7.419	0.934	0.525	0.169	0.078	0.405	0.286	0.073	0.325	69.54

**Table 2:** Performance comparison under different training methods.

Predictive method	MSE	RMSE	NMSE	SSE	MAPE
MO-BP	0.5245	0.7242	0.0328	26.2236	0.8617
VL-BP	0.4987	0.7062	0.0320	24.9357	0.8232
LM-BP	0.0558	0.2362	0.0107	2.7897	0.2570
CSA-BP	0.0390	0.1976	0.0004	1.9521	0.1897
SCSA-BP	0.0146	0.1207	0.0003	0.7282	0.0993
